# The Relationship between Hematological Markers of Systemic Inflammation (Neutrophil-To-Lymphocyte, Platelet-To-Lymphocyte, Lymphocyte-To-Monocyte Ratios) and Ultrasound Disease Activity Parameters in Patients with Rheumatoid Arthritis

**DOI:** 10.3390/jcm9092760

**Published:** 2020-08-26

**Authors:** Bożena Targońska-Stępniak, Robert Zwolak, Mariusz Piotrowski, Krzysztof Grzechnik, Maria Majdan

**Affiliations:** 1 Department of Rheumatology and Connective Tissue Diseases, Medical University of Lublin, 20-059 Lublin, Poland; zwolakr@wp.pl (R.Z.); mariusz_piotrowski@yahoo.com (M.P.); maria.majdan@gmail.com (M.M.); 2 Department of Rheumatology and Connective Tissue Diseases, Independent Public Teaching Hospital No 4, 20-954 Lublin, Poland; krzysiek.grzechnik@gmail.com

**Keywords:** NLR, PLR, LMR, rheumatoid arthritis, ultrasound, disease activity

## Abstract

Background: An accurate measurement of disease activity is essential for the appropriate management of a patient with rheumatoid arthritis (RA). Hematological markers of systemic inflammation (Neutrophil-to-Lymphocyte (NLR), Platelet-to-Lymphocyte (PLR) and Lymphocyte-to-Monocyte (LMR) ratios) are reported to be novel, sensitive measures of inflammatory response, in addition to conventional markers (erythrocyte sedimentation rate (ESR), C-reactive protein (CRP), Disease Activity Score (DAS28)). The goal of the study was to assess the relationship of NLR, PLR, and LMR with ultrasonography (US) parameters of disease activity in RA patients. Methods: The study group consisted of 126 consecutive RA patients (100 women, 26 men). The following assessments were performed: joint counts, DAS28, complete blood cell counts, ESR, CRP, and US of 24 small joints. Results: NLR and PLR were significantly positively correlated with all US parameters of disease activity (Grey Scale US, Power Doppler US, and Global scores). The mean values of NLR and PLR were significantly higher in patients with poor prognostic factors: moderate/high vs. low disease activity (NLR: *p* < 0.001; PLR: *p* = 0.007), anti-CCP positive vs. anti-CCP negative (NLR: *p* = 0.01; PLR: *p* = 0.006). In multiple regression tests, significant correlations were confirmed for: NLR and DAS28 (*p* = 0.04), and CRP (*p* = 0.001); PLR and Power Doppler US (*p* = 0.04), and ESR (*p* = 0.02). No correlation was found for LMR. Conclusion: NLR and PLR are associated with US disease activity parameters and may serve as reliable, inexpensive markers, with prognostic significance in RA.

## 1. Introduction

Rheumatoid arthritis (RA) is a chronic autoimmune disease, characterized by progressive, symmetric polyarthritis, leading to the irreversible destruction and deformities of joints. Inflammation is the key component of RA pathology. In an active phase of RA, inflammatory markers are generally high and correlate with high disease activity [1,2]. An appropriate assessment of the disease activity and inflammation intensity is essential for customizing the treatment strategy. The therapeutic target is a sustained remission, or at least low disease activity, which should prevent joint destruction, improve quality of life, and reduce comorbidity risks [3]. The disease activity is usually assessed by the Disease Activity Score of 28 joints (DAS28) system, which is calculated by the number of tender joints, number of swollen joints, patient assessment in visual analogue scale (VAS), and erythrocyte sedimentation rate (ESR) or C-reactive protein (CRP) concentration. These measures may not convey sufficiently reliable results in specific clinical situations [3,4]. Recently, the clinical assessment of RA activity may be verified by ultrasound (US) imaging of affected joints; however, this technique requires the proper equipment and skills. Therefore, there is an ongoing need to develop measurable biomarkers that could facilitate the proper assessment of RA activity [2].

Chronic inflammatory diseases are characterized by immune system dysregulation and persistent inflammation, which adversely affects the hematopoietic system due to the production of cytokines, antibodies, immune complexes, growth factor deficiencies, and toxicities related to medications [4]. The immune system elements—neutrophils, lymphocytes, monocytes, and platelets—have a significant role in the control of systemic inflammation and undergo changes secondary to an inflammatory state.

Recently, hematological parameters (Neutrophil-to-Lymphocyte ratio (NLR), Platelet-to-Lymphocyte ratio (PLR) and Lymphocyte-to-Monocyte ratio (LMR)) have come into use as markers of systemic inflammation. They have been reported to be highly sensitive measures of inflammation in the field of oncology (lung cancer, esophageal cancer, gastric cancer, and endometrial cancer), cardiology, nephrology, diabetes, infectious diseases, and autoimmune rheumatic diseases [5,6,7,8,9,10,11,12,13]. Increased NLR and PLR have been reported to be associated with cancer [5], diabetes [7], and psoriasis [9]. LMR has been reported as a biomarker for infectious diseases, as a result of the balance between effector and host [1]. Elevated NLR was found as an independent predictor for in-hospital and long-term mortality in patients with acute heart failure [8], and NLR and PLR are candidates for predicting renal outcomes in patients with rapidly progressive glomerulonephritis [13].

Recent studies have reported that NLR and PLR were significantly higher in patients with RA than in healthy control subjects [1,2,4,10,14]. Additionally, NLR and PLR were positively correlated with the disease activity and inflammatory parameters, as well as predicted treatment responses in patients with RA [1,2,4,15]. An association was also found between LMR and disease activity in RA patients [12]. The purpose of this study was to assess the associations between NLR, PLR, LMR, and US parameters, as well as with other (clinical and laboratory) markers of the disease activity in patients with RA.

## 2. Materials and Methods

### 2.1. Study Population

The study group consisted of 126 RA patients, hospitalized in the Department of Rheumatology and Connective Tissue Diseases, Medical University of Lublin. All the patients fulfilled the American College of Rheumatology (ACR)/European League Against Rheumatism (EULAR) classification criteria for RA [16]. This study was conducted in accordance with the Declaration of Helsinki of 1975, revised in 2013. The design of the study was approved by the Ethics Committee of the Medical University of Lublin (approval number KE-0254/319/2016, obtained before undertaking the research). Informed consent was obtained from each patient after an adequate explanation of the study design, prior to their inclusion in the study.

### 2.2. Clinical and Laboratory Findings

Baseline demographic and clinical data were collected through medical interviews and reviews of medical history and records.

A physical examination was performed, including a tender joint count (TJC) and swollen joint count (SJC). The disease activity of RA was determined using the 28 joints Disease Activity Score system (DAS28), calculated with TJC, SJC, ESR, and patient global assessment (PGA) in visual analogue scale (VAS) [17]. The cut point for low disease activity was DAS28 ≤ 3.2.

The ability to perform daily activities was assessed using a modified Health Assessment Questionnaire (M-HAQ), with range 0–3 (score 0 representing no impairment of function) [18].

An erosive form of RA was identified in patients who had erosions on joint surfaces of bones in X-rays of hands and/or feet, as assessed according to the Sharp/van der Heijde score by a trained radiologist [19].

Blood was collected after overnight fasting. Routine laboratory data determined for all patients included: complete blood cell count (CBC), ESR, and CRP. NLR was calculated as the ratio of peripheral blood neutrophil count to lymphocyte count; PLR was calculated as the ratio of peripheral blood platelet count to lymphocyte count; LMR was calculated as the ratio of peripheral blood lymphocyte count to monocyte count.

### 2.3. Ultrasound Imaging of Joints

Ultrasound (US) imaging was performed, including 24 bilateral joints: wrists (radio-carpal, midcarpal), metacarpophalangeal (MCP), hand proximal interphalangeal (PIP), thumb interphalangeal (IP), and fifth metatarsophalangeal (MTP) joints. An examination was performed using a machine (MyLab25 Gold, Esaote, Genova, Italy) equipped with an 18 MHz broadband high frequency linear array transducer for synovium hypertrophy and effusion (grey scale ultrasound, GSUS), with identical Power Doppler settings (PRF—pulse repetition frequency—700 Hz; gain was increased to the maximum level, not generating random noise with the lowest wall filters).

We applied the most frequently used approach for scoring synovitis:

1. Semi-quantitative grey scale (GS) for grading synovial hypertrophy (0–3) in each joint:
  - Grade 0: normal joint with no synovial hypertrophy;
  - Grade 1: synovial hypertrophy up to the level of the horizontal line connecting the bone surfaces of an examined joint;
  - Grade 2: synovial hypertrophy extending beyond the joint line but with the upper surface flat to the underlying bones;
  - Grade 3: synovial hypertrophy extending beyond the joint line but with the upper surface convex to the underlying bones.
2. Power Doppler ultrasound (PDUS) semi-quantitative scale (0–3) in each joint:
  - Grade 0: no Doppler activity;
  - Grade 1: up to three single Doppler spots, or up to one confluent spot and two single spots, or up to two confluent spots;
  - Grade 2: greater than grade 1 but <50% Doppler signals in the total GS background;
  - Grade 3: greater than grade 2 and >50% Doppler signals of the GS background [20].

Images were acquired according to the EULAR recommendations with a longitudinal scan, obtained using either a dorsal view (wrists), a dorsal or volar (plantar) view (MCPs, PIPs, thumb Ips, and fifth MTPs) for both GSUS and PDUS scores [21]. The highest score from the dorsal or volar (plantar) view of MCPs, PIPs, thumb IPs, and fifth MTPs was taken for a statistical calculation. Then, we calculated the GSUS and PDUS scores by summing the scores obtained as a result of the assessment of individual joints (range 0–72). The global score was calculated by summing the GSUS and PDUS scores from all the examined joints (range 0–144) [22].

### 2.4. Statistical Analysis

The results were presented as a mean (standard deviation, SD), or a number (%) and a range (minimum and maximum value). The results were tested for normality by Kolmogorov–Smirnov’s test. In order to compare subgroups of patients, Student’s t-test, or a nonparametric Mann–Whitney U test, were used. The correlation between quantitative variables was assessed by Spearman’s or Pearson’s correlation test. A multiple linear regression test was performed introducing variables that showed statistically significant association with NLR, PLR, and LMR. The independent variables used in the regression analysis included: US scores (GSUS, PDUS, and Global), laboratory parameters (ESR, CRP, Hb, and WBC) and clinical parameters of RA activity (TJC, SJC, DAS28, PGA, duration of morning stiffness, and M-HAQ). For all tests, *p*-values < 0.05 were considered significant.

## 3. Results

### 3.1. Demographic and Disease-Related Variables in RA Patients

The study group consisted mostly of women (almost 80%). The vast majority of patients had an erosive form of RA, and over 80% were seropositive (RF-IgM and/or ACPA). Extra-articular symptoms (rheumatoid nodules, sicca syndrome, interstitial lung disease, and vasculitis) in the course of the disease were observed in over 50% of patients. Low disease activity (DAS28 ≤ 3.2) at the time of examination was found in about 35% of patients.

At the time of examination, conventional synthetic disease modifying anti-rheumatic drugs (csDMARDs) were used in 124 patients and included methotrexate (MTX) in 109 (86.5%) patients (dose 10–25 mg/week, in monotherapy or combination), leflunomide (LEF) 9 (7.1%), hydroxychloroquine (HCQ) or chloroquine (CQ) 27 (21.4%), and sulfasalazine (SS) 2 (1.6%). Low-dose glucocorticoid (GC) therapy (prednisone ≤ 10 mg/day) was used in 83 (65.9%) patients.

The clinical characteristics of patients with RA are presented in Table 1.

### 3.2. Relationship between NLR, PLR, LMR, and Ultrasound Disease Activity Markers

**Figure 1:**
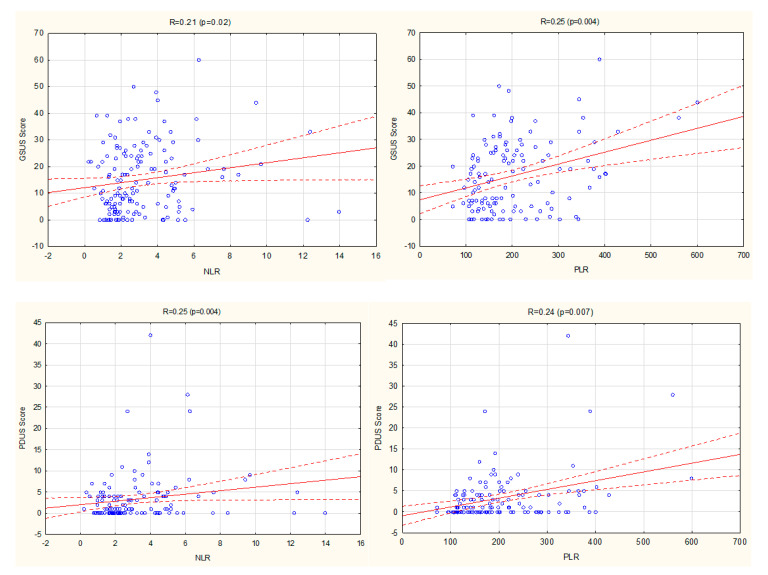

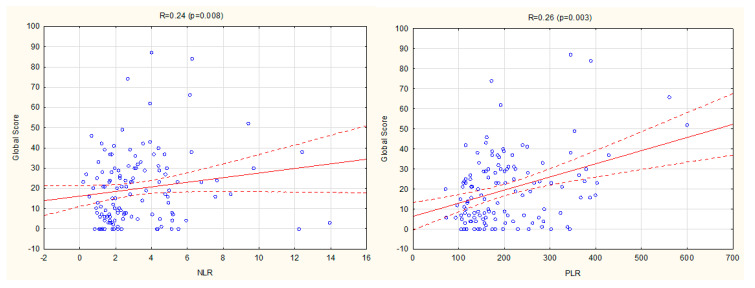
Correlations between NLR, PLR, and US scores (GSUS, PDUS, Global score) in RA patients. GSUS, Grey Scale Ultrasound; NLR, neutrophil–lymphocyte ratio; PDUS, Power Doppler ultrasound; PLR, platelet–lymphocyte ratio; RA, rheumatoid arthritis; US, ultrasound.

Positive correlations were found between both NLR and PLR, with the following US scores: GSUS score, PDUS score, and Global Score (Figure 1). There were no such correlations between LMR and US parameters.

In the multiple linear regression analysis, a significant positive association was confirmed for PLR with PDUS score (b = 3.95, *p* = 0.04).

### 3.3. The Relationship between Ultrasound and Other Disease Activity Markers

Significantly positive associations were found between GSUS, PDUS, global scores, and all the following clinical parameters of RA activity: TJC, SJC, DAS28, PGA, duration of morning stiffness, ESR value, and CRP concentration (Table 2).

In the multiple linear regression analysis, significant positive associations were confirmed for: GSUS with SJC (b = 1.79, *p* < 0.001); PDUS with SJC (b = 0.66, *p* < 0.001); global score with SJC (b = 2.81, *p* < 0.001).

### 3.4. The Relationship between NLR, PLR, LMR, and Clinical Disease Activity Markers

Positive correlations were found between NLR and the following parameters: DAS28, TJC, SJC, PGA, the duration of morning stiffness, and M-HAQ (Table 2). Positive correlations were found between PLR and the following parameters: DAS28, TJC, SJC, PGA, and the duration of morning stiffness (Table 2). There were no correlations between LMR and clinical disease activity parameters (Table 2).

In the multiple linear regression analysis, a significant positive association was confirmed for NLR and DAS28 (b = 0.62, *p* = 0.04).

### 3.5. The Relationship between NLR, PLR, LMR, and Laboratory Disease Activity Markers

Positive correlations were found between NLR and the following parameters: CRP, ESR, and white blood cell (WBC) count (Table 2). Positive correlations were found between PLR and CRP, and ESR. Negative correlations were found with hemoglobin concentration (Table 2). A positive correlation was found between LMR and CRP (Table 2).

In the multiple linear regression analysis, significant positive associations were confirmed for NLR with CRP (b = 0.05, *p* = 0.001) and WBC count (b = 0.31, *p* = 0.002), as well as for PLR with ESR (b = 0.83, *p* = 0.02).

### 3.6. A Comparison of NLR, PLR, and LMR in Certain Groups of RA Patients

**Figure 2:**
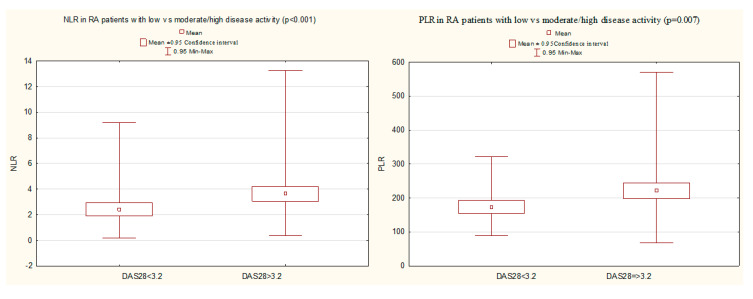
A comparison of NLR and PLR in RA patients with low vs. moderate/high disease activity (Mann–Whitney U test). DAS28, Disease activity score in 28 joints; NLR, neutrophil-to-lymphocyte ratio; PLR, platelet-to-lymphocyte ratio; RA, rheumatoid arthritis.

The group of RA patients with moderate/high disease activity (DAS28 > 3.2), compared with patients with low disease activity (DAS28 ≤ 3.2), was characterized by significantly higher values of NLR (3.85 (2.66) vs. 2.40 (1.46), *p* < 0.001) and PLR (221.56 (106.17) vs. 173.16 (63.21), *p* = 0.007) (Figure 2).

**Figure 3:**
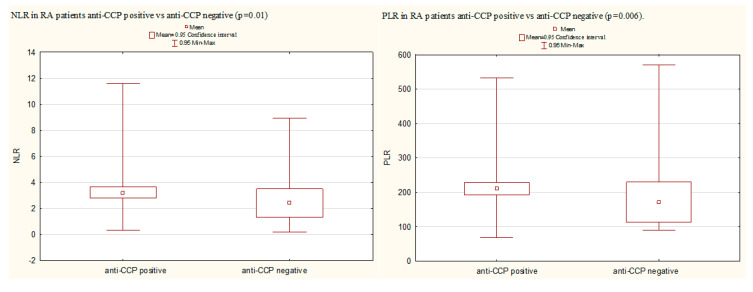
A comparison of NL and PLR in RA anti-CCP positive patients vs. anti-CCP negative patients (Mann–Whitney U test). Anti-CCP, anti-citrullinated peptide/protein antibodies; NLR, neutrophil-to-lymphocyte ratio; PLR, platelet-to-lymphocyte ratio; RA, rheumatoid arthritis.

Significantly higher mean values of NLR and PLR were observed in anti-CCP positive patients compared with anti-CCP negative patients (respectively, NLR: 3.41 (2.28) vs. 2.46 (2.13), *p* = 0.01 and PLR: 210.15 (91.36) vs. 171.28 (117.81, *p* = 0.006) (Figure 3). No significant differences were found in the LMR value.

## 4. Discussion

Our study showed that, in patients with RA, hematological markers of systemic inflammation (NLR and PLR) were significantly positively correlated with US parameters of the disease activity (GSUS, PDUS, and global scores). We also found significant positive associations of NLR and PLR with both clinical (DAS28, SJC, and TJC) and laboratory (CRP and ESR) disease activity markers.

The mean NLR and PLR were significantly higher in patients with poor prognostic factors according to the EULAR recommendations (moderate/high disease activity, presence of anti-CCP antibodies, or combination of the mentioned factors), compared to patients without prognostically unfavorable factors [3].

Both methods (hematological markers and US of joints) are effective and complementary in the assessment of disease activity in RA. From a clinical point of view, the association between hematological parameters and inflammatory disease activity markers is very important because hematological markers are quite cheap and widely available and may be assessed in every patient. Recently, the clinical assessment of RA activity is often verified by US imaging, which shows the extent and severity of local inflammation in joints. However, an adequate US assessment requires the proper equipment, as well as the good skills and experience of an investigator.

Our results are consistent with the data in the literature. The significant correlation between NLR, PLR, and DAS28 was reported, resulting in lower NLR and PLR values in RA patients with remission (DAS28 < 2.6). The authors concluded that NLR and PLR could be used to assess disease activity in patients with RA [1]. In the recent meta-analysis of studies reporting on NLR and PLR in RA, it was found that the two markers were significantly associated with the presence of RA and were higher in RA patients when compared to the controls [14].

It was also reported that NLR might serve as a less expensive and effective measure of inflammation in RA, with the efficacy comparable to that of CRP. The NLR cut-off value of 1.4 classified patients in deep remission with 90% specificity and 24% sensitivity [2]. In another study, NLR significantly correlated with disease activity indices in recent onset RA. The predictors of NLR included DAS28, PDUS, and ESR. NLR was significantly higher in patients with high disease activity than in those with moderate and low disease activity [4]. The common inflammatory parameters (ESR and CRP) are good indicators of systemic inflammation, but they may not reflect localized, subclinical inflammation. Therefore, more sensitive tools are necessary to assess persisting inflammation, especially in RA patients who have achieved low disease activity or remission according to the clinical assessment.

In recent years, an increasing use of US imaging has been observed, as a tool for the assessment of inflammatory arthritis, particularly in RA patients. Both US parameters, GS and PD, have been shown to be sensitive to—and even predictive of—destructive arthritis and radiographic structural damage. GS is useful in monitoring morphological changes (effusion and synovial hypertrophy), and PD is useful in monitoring the hypervascularity of synovium (neoangiogenesis). However, there is still a debate on how to grade the detected changes, and how to monitor US inflammatory symptoms [20]. Recently, it has been reported that swollen joints were strongly associated with US-detected synovitis (GS/PD score), reflecting ongoing inflammation, while this association was not found for tender joints [23]. Our results are consistent with these data—the significant correlations were confirmed between all the assessed US scores and SJC. The synovial inflammation detected by US is associated with the infiltration of neutrophils and lymphocytes.

NLR represents two compartments of the immune system: neutrophil representing the innate system, and lymphocyte representing the adaptive system [2]. Neutrophils are at the front line of the defense system. They are responsible for the production of lytic enzymes, free oxygen radicals, and cytokines [4]. Lymphopenia in peripheral blood might be the result of the persistent accumulation of lymphocytes at the sites of inflammatory joints and might be due to the increased apoptosis of lymphocytes in RA patients. Early apoptotic markers are high in lymphocytes which might be associated with autoimmunity and results in lymphopenia [1,12]. Peripheral lymphopenia and the gradual increase in neutrophil count have been often noted with the progression of RA [2]. Reactive thrombocytosis is induced by chronic inflammation, cancers, or infectious diseases, by accelerating the production of thrombopoietin and cytokines [11]. Platelets also have an active role in inflammation, having regulatory effects on the immune system, and are involved in the production of cytokines [1]. As a result of changes caused by inflammation in neutrophils, lymphocytes and platelets, NLR, PLR, and LMR have turned into inflammatory markers. The reduced LMR might also be caused by a treatment with drugs such as methotrexate, leflunomide, or glucocorticoids [12]. Hematological inflammatory markers are easily available and reliable, as well as cost-accessible. Variability in NLR has been reported in different races, due to the presence of other inflammatory conditions and lifestyle habits such as smoking [2].

Our study has some potential limitations. First, the relatively small number of patients included in the study; a higher number of patients could enable better statistical evaluation. Second, we did not explore the relationship of hematological markers with indices other than DAS28, such as CDAI or SDAI. Third, we could not exclude the influence of other concurrent medical conditions or smoking, which could potentially influence the value of hematological markers. Fourth, we could not exclude the influence of concomitant treatment (DMARDs or GC), because the study was performed in real-life RA patients.

Our study also has several strengths. First, to our best knowledge, it is the first study assessing the associations between hematological parameters of systemic inflammation and different US scores. Second, it has detailed characteristics of the patients, which were considered in all aspects of RA pathology. Third, patients were not selected for the study—they are real patients. Fourth, the same experienced physician performed all of US measurements, thereby eliminating any interpersonal variations. Fifth, all assessments performed are available on an outpatient basis.

## 5. Conclusions

The results of our study point to a significant relationship between NLR, PLR, and US disease activity parameters. We found that NLR and PLR correlate with parameters of RA activity, both laboratory (CRP and ESR) and clinical (DAS28, SJC, and TJC). Higher values of NLR and PLR were observed in RA patients with poor prognostic factors (moderate/high disease activity and positive anti-CCP antibodies). In this study, we showed that hematological parameters of systemic inflammation are significantly correlated with the US disease activity parameters. NLR and PLR may serve as reliable markers of RA systemic activity; however, they may also serve as markers of local inflammation in the joints affected by RA.

## Figures and Tables

**Table 1:** Characteristics of 126 patients with RA.

Data	Results (Mean (SD) (Range) or Number (%))
Age, years	53.8 (11.9) (20–82)
Gender, female/male	100 (79.4)/26 (20.6)
RA related variables:	
Disease duration, years	14.0 (10.9) (1–45)
Positive RF-IgM	110 (87.3)
Positive anti-CCP	104 (82.5)
Extra-articular manifestations	70 (55.6)
Erosions (hands/feet)	107 (84.9)
M-HAQ	1.4 (0.7) (0–3.5)
Current conventional DMARD	124 (98.4)
Current biological treatment	62 (49.2)
Current glucocorticoid use	83 (65.9)
Laboratory results:	
Hemoglobin, g/dL	12.8 (1.4) (9.4–16.1)
White blood cells	6.9 (2.4) (3.2–14.6)
Platelets, 10^9^/L	289.2 (78.2) (124–505)
Neutrophils, 10^9^/L	4.6 (2.4) (1.1–12.9)
Lymphocytes, 10^9^/L	1.6 (0.6) (0.6–4.2)
Monocytes, 10^9^/L	0.4 (1.8) (0.1–1.5)
NLR	3.34 (2.4) (0.5–14.0)
PLR	204.5 (95.9) (71.2–600)
LMR	4.4 (2.5) (1.2–15.8)
CRP, mg/L	15.0 (18.7) (0.6–85.0)
ESR, mm/h	35.3 (28.3) (2–120)
Clinical parameters of RA activity:	
TJC	5.5 (5.5) (0–21)
SJC	3.7 (4.4) (0–19)
PGA (VAS), mm	37.6 (26.0) (0–98)
Morning stiffness, minutes	56.1 (63.1) (0–300)
DAS28	4.19 (1.8) (0.7–7.8)
Low disease activity (DAS28 < 3.2)	45 (35.7)
GSUS score (hypertrophy)	16.5 (13.2) (0–60)
PDUS score	3.3 (5.8) (0–42)
Global score	19.8 (17.6) (0–87)

Data are presented as mean (SD) (range) or number (%). Anti-CCP, anti-cyclic citrullinated peptide antibodies; CRP, C-reactive protein; DAS28, disease activity score in 28 joints; ESR, erythrocyte sedimentation rate; GSUS, Grey Scale Ultrasound; LMR, lymphocyte–monocyte ratio; M-HAQ–modified health assessment questionnaire; NLR, neutrophil–lymphocyte ratio; PDUS, power doppler ultrasound; PGA, patient global assessment; PLR, platelet–lymphocyte ratio; RA, rheumatoid arthritis; RF-IgM, IgM rheumatoid factor; SJC, swollen joints count TJC, tender joint count; VAS, Visual Analogue Scale.

**Table 2:** Statistical results (R Spearman) of associations between different disease activity parameters and inflammatory markers in RA patients.

Data/*p* Value/R	CRP	ESR	DAS28	TJC	SJC	PGA	Morning Stiffness	M-HAQ	WBC Count	Hb
NLR	<0.001	<0.001	<0.001	0.004	0.001	0.003	0.006	0.03	<0.001	NS
	0.5	0.37	0.37	0.26	0.3	0.26	0.27	0.2	0.54	
PLR	<0.001	<0.001	<0.001	0.003	0.002	0.02	0.04	NS	NS	0.005
	0.31	0.35	0.35	0.26	0.28	0.22	0.21			−0.25
LMR	0.02	NS	NS	NS	NS	NS	NS	NS	NS	NS
	−0.21									
GSUS score	0.02	0.006	<0.001	0.007	<0.001	<0.001	0.05	NS	NS	NS
	0.21	0.25	0.36	0.24	0.47	0.34	0.2			
PDUS score	<0.001	0.002	<0.001	<0.001	<0.001	<0.001	<0.001	0.02	NS	NS
	0.31	0.27	0.42	0.36	0.6	0.39	0.38	0.22		
Global score	0.007	0.003	<0.001	0.002	<0.001	<0.001	0.01	0.04	NS	NS
	0.24	0.27	0.4	0.28	0.52	0.36	0.25	0.2		

CRP, C-reactive protein; DAS28, disease activity score in 28 joints; ESR, erythrocyte sedimentation rate; GSUS, Grey Scale Ultrasound; LMR, lymphocyte–monocyte ratio; Hb, hemoglobin; M-HAQ, modified health assessment questionnaire; NLR, neutrophil–lymphocyte ratio; PDUS, power doppler ultrasound; PGA, patient global assessment; PLR, platelet–lymphocyte ratio; RA, rheumatoid arthritis; RF-IgM, IgM rheumatoid factor; SJC, swollen joints count TJC, tender joint count; VAS, Visual Analogue Scale; WBC, white blood cell count.
